# In Search of Resistance Against Fusarium Ear Rot: Ferulic Acid Contents in Maize Pericarp Are Associated With Antifungal Activity and Inhibition of Fumonisin Production

**DOI:** 10.3389/fpls.2022.852257

**Published:** 2022-04-08

**Authors:** Javier Martínez-Fraca, M. Eugenia de la Torre-Hernández, Max Meshoulam-Alamilla, Javier Plasencia

**Affiliations:** Departamento de Bioquímica, Facultad de Química, UNAM, Mexico City, Mexico

**Keywords:** maize, Fusarium ear rot (FER), fumonisin, ferulic acid, *Fusarium verticillioides*

## Abstract

*Fusarium verticillioides* is a fungal pathogen of maize that causes seedling blight, stem rot, and Fusarium ear rot. Fungal infestation of maize kernels and ears affects grain quality from the ensuing mycotoxin buildup. Among the mycotoxins produced by *F. verticillioides*, fumonisins accumulate to high levels in *Fusarium*-infected maize kernels, fumonisin B1 (FB1) being the most abundant in naturally infected maize. Achieving resistance to Fusarium ear rot has been challenging, as various environmental factors facilitate fungal infection. Among the maize grain components that contribute to resistance to *F. verticillioides* infection, the pericarp is the first barrier faced by the fungus and thus plays a key role. Phenolic acids are major constituents of maize pericarp, of which ferulic acid (FA) is the predominant molecular species. In this work, we explored the relationship between FA levels, fungal infection, and FB1 production in 51 maize genotypes and whether the antioxidant activity of FA might play a role. We confirmed that FA is a major component of the seed pericarp, whose levels as bound FA varied between 4.5 and 26.3 mg/g across maize genotypes. We selected two pools of five maize varieties, with contrasting FA contents: low FA (LFA; 6.14 ± 0.40 mg/g) and high FA (HFA; 15.49 ± 1.31 mg/g). *In vitro*, HFA extracts inhibited fungal growth with effects comparable to FA concentrations in the 0.25–0.50 mM range. We also established a kernel assay to study *F. verticillioides* colonization and FB1 production in the LFA and HFA genotypes. Fungal colonization was significantly lower in HFA genotypes relative to LFA genotypes, based on ergosterol levels. Moreover, FB1 production was also inhibited in the HFA genotypes. Importantly, the antioxidant activity of maize pericarp extracts was associated with FA contents, with HFA extracts exhibiting a greater antioxidant activity than LFA extracts. Overall, our results highlight the role of FA and its antioxidant activity on resistance to Fusarium ear rot and provide the basis of a phenotypic trait that can be deployed for breeding selection.

## Introduction

*Fusarium* spp. are ubiquitous fungal pathogens in maize (*Zea mays*) and cause seedling blight, stalk rot, as well as ear and kernel rot. Within this genus, *Fusarium verticillioides* (Sacc.) Nirenberg, *F. proliferatum* (Matsush.) Nirenberg, and *F. subglutinans* (Wollenw. and Reinking) are relevant species responsible for Fusarium ear rot (abbreviated FER hereafter) (Munkvold, 2003; Mesterházy et al., 2012). Colonization of maize stalks and ears is facilitated when the European corn borer moth (*Ostrinia nubilalis*) deposits its eggs on the leaves, which provide a food source for the emerging larvae and offer an entrance point to the fungus for successful infection. At the silking stage, the fungus can also enter through the stylar canal and reach the developing kernels (Sobek and Munkvold, 1999; Duncan and Howard, 2010; Blacutt et al., 2018).

Direct yield losses associated with FER are compounded by the several mycotoxins *F. verticillioides* produces. Of these, fumonisins are the major metabolites produced by these fungal species, and various surveys have observed incidence rates above 50% in maize grain samples, with a mean mass of 0.36 mg/kg and a maximum value reaching over 10 mg/kg (Marin et al., 2013). In a survey of maize fields in Northern Mexico, total fumonisin contents ranged from 0.5 to 6.8 mg/kg (Cortez-Rocha et al., 2003), which might reflect on high mycotoxin intake. Corn is consumed mainly in form of *tortillas* and other nixtamalized products, and daily consumption per person has been estimated to be between 100 and 150 g/day. Recent surveys in eastern Mexico have found a high percentage (77.5–87.5%) of samples contaminated with FB1, with levels ranging from 9.5 to 689 μg/kg (Wall-Martínez et al., 2019).

Fumonisins are classified as probable carcinogens (Group 2B) by the International Agency for Research on Cancer (IARC) (Ostry et al., 2017). The maximum allowed limits for total fumonisin consumption by humans were set to 4 mg/kg in maize products in the United States by the Food and Drug Administration (2001), while the European Union uses stricter criteria with a maximum content of 1 mg/kg in maize-based foods (Knutsen et al., 2018).

Fusarium ear rot resistance is a quantitative trait determined by multiple genes (Mesterházy et al., 2012; Lanubile et al., 2017). Characteristics such as husk tightness are associated with FER resistance and lower mycotoxin contamination (Butrón et al., 2006). Pericarp thickness and composition are linked to FER tolerance; a 2-year field trial showed that Dent corn hybrids with high or intermediate resistance to *F. verticillioides* have a thicker pericarp compared to susceptible genotypes (Hoenisch and Davis, 1994). Besides pericarp thickness, its chemical composition has been associated with resistance to *F. verticillioides* infection and fumonisin B1 (FB1) accumulation. In field trials, maize genotypes with high kernel phenylpropanoid contents showed lower disease severity and fumonisin contamination in grains than those with lower levels of these metabolites, ferulic acid (FA) being the major phenolic accumulating in this tissue (Sampietro et al., 2013). Most of the FA is bound to cell wall polysaccharides and can dimerize to form crosslinks between arabinoxylans chains, and at least four forms of dehydrodimers of FA have been described in maize grains (Bily et al., 2003).

Phenolic acids inhibit the growth of both filamentous and yeast fungi. Multiple studies have addressed this question, but no clear mode of action has been proposed. Most studies have been conducted with the opportunistic human pathogen *Candida albicans*, and evidence indicates that cell membrane integrity might be affected, either directly or by inhibiting ergosterol biosynthesis (Teodoro et al., 2015). Moreover, several phenolic compounds have been shown to induce apoptotic pathways in *Candida*, thereby exerting their antifungal activity (Zore et al., 2011). Most of these studies have employed various phenolics, such as isoquercetin, curcumin, lariciresinol, and eugenol, or plant extracts containing high levels of FA (Teodoro et al., 2015). In filamentous fungi, the effects of phenolic acids on fungal development and mycotoxin production have been studied in other genera. In *Aspergillus* sp., a concentration of 1 mM FA inhibits growth by 30% and aflatoxin production by 50% (Holmes et al., 2008). Other phenolic compounds vary in their effects; for example, salicylic acid (1 and 5 mM) does not affect *Aspergillus* growth, whereas similar concentrations of thymol and cinnamic acid cause a 50% to 70% growth inhibition (Kim et al., 2006).

Moreover, available data in the literature can be contradictory about the effective phenolic acid concentration required to inhibit fungal growth and fumonisin production. For instance, Ferrochio et al. (2013) reported that high concentrations (10–25 mM) of FA significantly reduce the growth rate of *F. verticillioides* and *F. proliferatum*, as well as fumonisin production. However, lower FA doses (1 mM) have the opposite effect and increase toxin production. By contrast, Beekrum et al. (2003) showed that caffeic acid, FA, and vanillic acid at 1 μg/mL (roughly equivalent to 0.005 mM) inhibit FB1 production by 90% relative to untreated controls.

The antioxidant capacity of phenolic acids and other metabolites in maize seeds might be responsible for inhibiting fumonisin production; 0.1 mM α-tocopherol inhibits 30–70% of fumonisin production, while 1 mM FA suppresses 50–70% of toxin production. Studies *in planta* with three maize genotypes with different susceptibility to FER showed a close association between resistance and fumonisin accumulation with FA levels in immature kernels (Picot et al., 2013). Although the contribution of maize metabolites with antioxidant activity has been reported, the exact mechanism by which they confer resistance against fungal infection is still unknown; in addition, the distribution of these phenolic compounds varied among the seed tissues (Das and Singh, 2015).

Because the pericarp is the first chemical and physical barrier facing a fungal infection, we studied the contribution of FA contents in this tissue toward the inhibition of *Fusarium* growth, as well as FB1 production. We show here that levels of bound FA vary significantly across maize genotypes. We performed *in vitro* experiments and a kernel assay to study the effects of pure FA and a methanolic pericarp extract on *F. verticillioides* growth and FB1 accumulation, as well as its antioxidant activity.

## Materials and Methods

### *Fusarium verticillioides* Strain and Plant Materials

The *F. verticillioides* (MY3) strain employed in this study was isolated from a maize ear (Sánchez-Rangel et al., 2005) and was classified as a high-FB1 producer among our strain collection (Galeana-Sánchez et al., 2017). Fifty maize varieties (100-g samples) were provided by the germplasm bank at the International Maize and Wheat Improvement Center (CIMMYT), located in El Batán, Mexico (Supplementary Table 1). The maize genotypes included in this study comprised a wide range of gene pools, mainly advanced inbred lines developed to improve maize productivity and tolerance against multiple stress factors in tropical/subtropical production environments. All maize genotypes had either white or yellow kernels. The pedigree of each genotype is given in Supplementary Table 2. The remaining genotype (landrace *Chalqueño*) was obtained from a local farmer in Chalco, Mexico.

### Ferulic Acid Extraction and Analysis

Extraction procedures were adapted from López-Martínez et al. (2009). A kernel subsample (15–20 g) of each maize genotype was immersed in 50 mL of deionized water for 2 h at room temperature. The water was drained, and the pericarp was peeled manually from each kernel using a scalpel or a single-edge blade. The pericarp was blotted onto paper towel and dried on filter paper at room temperature for 48 h. The dry pericarp tissue was cut with a blade to obtain smaller pieces. For bound FA analysis, three 100-mg pericarp subsamples from each maize genotype were weighed and ground in liquid nitrogen. The powder was extracted with 2 mL of a 2 M sodium hydroxide solution for 2 h at room temperature. The solution was acidified to reach a pH of 2 with concentrated HCl, and phenolic acids were extracted with ethyl acetate twice. The organic solvent was evaporated at 50°C under a stream of nitrogen, and the residue was dissolved in a methanol:water mixture (1:1, v/v). To analyze free FA contents in the maize pools, pericarp subsamples (600 mg each) were ground in liquid nitrogen and the powder was resuspended in 80% (v/v) ethanol and shaken for 10 min at 200 rpm on an orbital shaker. The extraction was repeated, and the supernatants were pooled for solvent evaporation under a stream of nitrogen. The residue was dissolved in a methanol:water mixture (1:1, v/v) and stored until analysis. FA was analyzed by high-performance liquid chromatography (HPLC) through separation on a C_18_ column (15 cm × 4.6 mm; 5 μm) using a mixture of methanol and 4% (v/v) acetic acid (1:3, v/v), at a flow rate of 1.2 mL/min. Analytes were detected with a UV/Vis detector (Shimadzu, model SPD-10AC) at 325 nm and quantified using FA (4-hydroxy-3-methoxycinnamic acid; Sigma-Aldrich) as primary standard. To obtain the pericarp extracts employed for antifungal activity, FB1 production assays, and antioxidant activity, a 50-g sample of each maize pool was used to detach the pericarp as described above. This amount of grains yielded approximately 2 g of dry pericarp, which was ground and subjected to alkaline hydrolysis for FA extraction by adjusting solvent volumes to the corresponding mass of dry powder. FA was quantified in these extracts to compare their biological activity to pure FA.

### Fungal Inoculum Preparation

The fungal strain was grown on Potato-Dextrose Agar (PDA) plates for 7–10 days at 28°C, and conidia were loosened by adding 7 mL of sterile water and shaking on an orbital shaker (150 rpm) for 90 min at room temperature. The conidia suspension was collected, and cell density was counted under the microscope in a Neubauer chamber. Dilutions of the spore suspension were prepared to adjust its concentration depending on the assay as described below.

### Antifungal Tests

Antifungal activity of FA and pericarp extracts was evaluated by inoculating 1–5 μL of the conidial suspension (1 × 10^6^ conidia) on a 5-mm sterile filter paper disk placed in the center of a half-strength PDA plate containing increasing concentrations of FA (0.1–10 mM). FA was dissolved in dimethyl sulfoxide (DMSO) for antifungal assays or in methanol for assays comparing its potency with the pericarp extracts, since the pericarp extracts used this solvent. Maximum solvent amount was 0.1% (v/v) for DMSO and 0.27% (v/v) for methanol. Plates were incubated at 28°C for 7–15 days, depending on the experiment, before radial growth was measured.

### Effect of Ferulic Acid on Fumonisin B1 Production

*In vitro* FB1 production was analyzed by inoculating 1.25 × 10^5^ conidia of *F. verticillioides* MY3 strain in 50-mL Erlenmeyer flasks containing 12 mL of GYAM medium (Bojja et al., 2004), containing FA or pericarp extracts. The flasks were incubated in the dark at 29°C for 12 days. A 2-mL aliquot of the medium was pipetted from each flask and transferred to an Eppendorf tube. The tubes were centrifuged at 4°C for 10 min at 12,100 rcf in an Eppendorf MiniSpin centrifuge, and the supernatant was transferred to a fresh tube and stored at –20°C until analyses. FB1 levels were analyzed by HPLC separation of the ortho-phthaldialdehyde (OPA) derivative using a Shimadzu RF10-AXL fluorescent detector (λ_*exc*_ = 335 nm; λ_*emm*_ = 440 nm) and quantified using pure FB1 (Sigma-Aldrich) as primary standard (Sydenham et al., 1992). The mycelium was retained in a Whatman filter paper, dried, and weighed to determine fungal biomass.

### Kernel Assay for Maize–*F. verticillioides* Interaction

To gather enough plant material for these assays, approximately 14 g of kernels was pooled for each of the five genotypes with the lowest bound FA contents (LFA: M10, M16, M17, M18, and M32) to reach a 70-g sample. The same procedure was performed for the genotypes with highest bound FA contents (HFA: M2, M13, M36, M39, and M40). Kernels (15 g) were immersed in a flask containing 100 mL of a 2.5% (w/v) sodium hypochlorite solution and were manually shaken for 2 min. The hypochlorite solution was decanted, and the seeds were washed three times with sterile water and blotted onto sterile filter paper. Kernels were inoculated by immersion in a conidial suspension (10^7^ conidia/mL) in sterile flasks and gently agitated (100 rpm) for 6 h at room temperature. The inoculated kernels were plated on 15-cm plates containing 1.2% (w/v) agar, and plates were incubated at 29°C for 24 h. Kernel samples were taken for ergosterol and FB1 analysis at 6, 12, and 24 h post inoculation (hpi).

### Ergosterol and Fumonisin B1 Levels in Inoculated Seeds

Ergosterol is an abundant lipid in fungal membranes that is used as a marker of fungal biomass in infected plant tissues (Mille-Lindblom et al., 2004). For ergosterol analysis, kernels sampled at 6, 12, and 24 hpi were immersed in 50 mL sterile water and shaken (200 rpm) for 10 min to detach the mycelia from the seed surface. The solution was centrifuged for 10 min at 3,400 rpm in a Beckman GS-6R centrifuge, the supernatant was discarded, and the pellet was lysed in 2 mL of methanol and 2 mL of 60% (w/v) KOH. The tubes were incubated for 1 h at 65°C and cooled to room temperature, and ergosterol was extracted twice with hexane. The solvent was evaporated to dryness under a stream of nitrogen, and the residue was dissolved in 0.2 mL methanol for HPLC analysis. Ergosterol standards (Sigma Chemical Co.) and samples were resolved in a 15-cm C18 column and analyzed using a SPD 10AV UV/VIS Shimadzu detector at 290 nm (Chiocchio and Matkovie, 2011).

FB1 production was assessed by taking kernel subsamples (6, 12, and 24 hpi) and immersing them in a 50-mL Falcon tube containing 40 mL of acetonitrile:water (9:1; v/v). The tubes were shaken for 2 h (250 rpm) at room temperature, and the solvent solution was filtered and transferred into glass tubes. The solvent was evaporated (50°C) under a stream of nitrogen, and the residue was dissolved in 200 μL of acetonitrile:water (1:1; v/v), stored at −20°C, and analyzed by HPLC as described above.

### Antioxidant Activity

Oxidized ABTS^+^ reagent was prepared by mixing 1.5 mL of 1.8 mM ABTS (Sigma Chemical Co.) with 27 μL of 0.63 mM potassium persulfate and allowed to react for 14 h before using it. The antioxidant activity of the pure phenolic acids and the pericarp extracts was evaluated by placing the respective ethanolic solutions (50 μL) in a 96-well plate followed by 100 μL of the ABTS^+^ solution. Absorbance (450 nm) was recorded on an Epoch, BioTek plate reader immediately after the addition of ABTS^+^ reagent. The percentage decrease of absorbance was calculated for each sample using the ABTS^+^ solution as blank (Re et al., 1999).

### Statistical Analysis

Student’s *t*-test, analysis of variance and Tukey’s test were performed using Statistix™ software (V.4, Analytical Software, Tallahassee, FL, United States). Bivariate analysis was performed with JMP Software, 14 PRO version (SAS Institute Inc., Cary, NC, United States).

## Results

### Wide Variation of Bound Ferulic Acid Content in Maize Seed Pericarp

**FIGURE 1:**
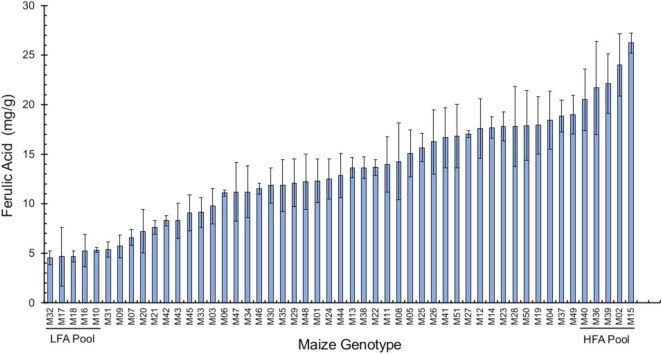
Bound ferulic acid levels in the pericarp of 51 maize genotypes. Pericarp tissue was manually peeled off from maize seeds. Dry pericarp (100-mg subsamples) was used for ferulic acid extraction and analysis. Data are shown as mean ± standard deviation (SD) (*n* = 3) of FA levels (mg/g pericarp tissue). Data were analyzed by analysis of variance (one-way ANOVA), and means were compared using Tukey’s test (*p* = 0.05). The genotypes comprising the pools with low ferulic acid (LFA) and high ferulic acid contents (HFA) are shown. Maize entry codes are given in Supplementary Table 1.

We analyzed bound FA levels in the seed pericarp of 51 different maize genotypes, which revealed a range from 4.5 to 26.3 mg/g pericarp, thus exhibiting a six-fold variation between extreme maize varieties (Figure 1 and Supplementary Table 1). We selected the two extremes of the FA distribution to categorize maize genotypes as low-FA (LFA) and high-FA (HFA) contents for further tests in their interaction with *F. verticillioides*. Using kernels from these two extreme pools, we determined that the low-FA pool accumulates an average 6.40 ± 0.40 mg bound FA/g pericarp, while the high-FA pool averaged 15.49 ± 1.31 mg bound FA/g pericarp, a difference that is significant (*p* < 0.01, Student’s *t*-test). We also quantified free FA in these samples and obtained a titer of 18.5 ± 2.8 μg/g for the LFA pool and 20.7 ± 2.6 μg/g for the HLA pool, which was not significantly different.

### Ferulic Acid Inhibits *Fusarium verticillioides* Radial Growth and Fumonisin Production

**FIGURE 2:**
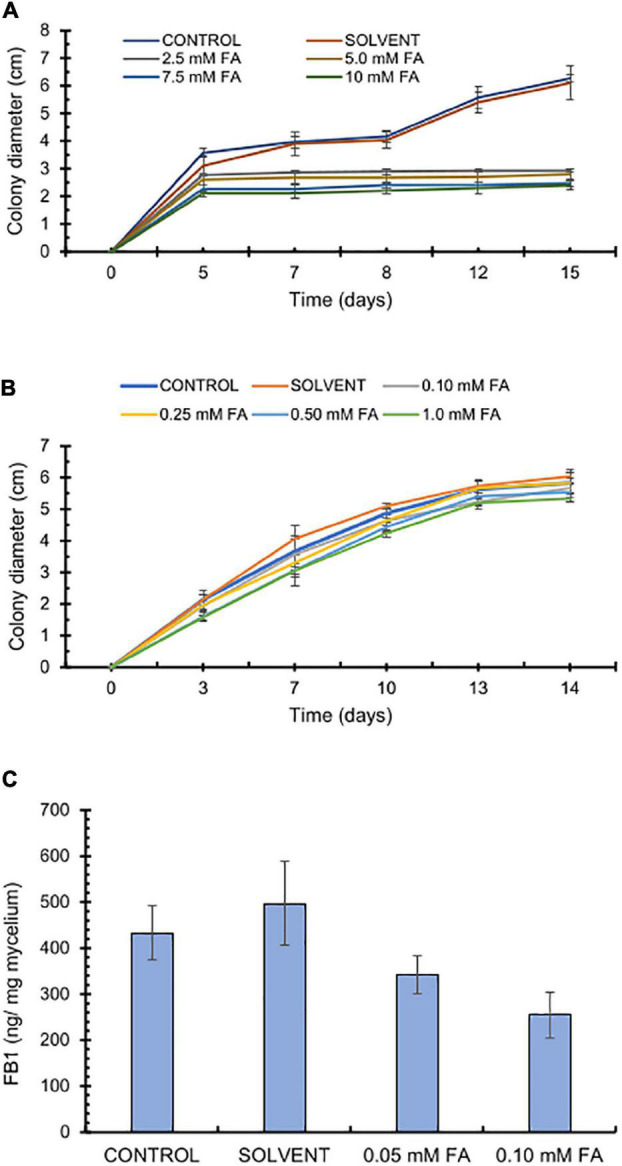
Effect of ferulic acid concentrations on *F. verticillioides* radial growth and fumonisin production. **(A)** Effects of high ferulic acid (FA) concentrations on fungal radial growth. 2 × 10^3^ conidia were inoculated in the center of PDA plates containing FA (2.5, 5, 7.5, or 10 mM). **(B)** Effects of low ferulic acid concentrations on fungal radial growth. 2 × 10^3^ conidia were inoculated in the center of PDA plates containing FA (0.1, 0.25, 0.5, or 1.0 mM). **(C)** Effect of ferulic acid on FB1 production. 1.25 × 10^5^ *F. verticillioides* conidia were inoculated in GYAM medium containing 0.05 or 0.1 mM FA. FB1 levels were analyzed 12 days post-inoculation by HPLC of the OPA fluorescent derivatives; values are means ± SD, and different lowercase letters indicate significant differences, as analyzed by ANOVA followed by Tukey’s comparison test (*n* = 4; *p* < 0.05).

The antifungal effects of FA have been documented against various phytopathogenic fungi; however, its activity varies depending on the strain and test conditions (Atanasova-Penichon et al., 2016), prompting us to test a wide concentration range. Accordingly, we tested high FA concentrations (2.5–10 mM) that inhibited fungal growth, possibly with fungistatic properties as we observed no further growth from day 5 onward (Figure 2A). We tested a second set of FA concentrations (0.1–1 mM), of which the two highest FA concentrations (0.5 and 1 mM FA) had a modest but significant effect (*p* < 0.05) on *F. verticillioides* growth, as determined by colony diameter, whereas the two lowest FA concentrations (0.1 and 0.25 mM FA) did not disturb fungal growth (Figure 2B). Because one of the goals of this work was to test how FA might influence FB1 production, we inoculated *F. verticillioides* in GYAM liquid medium containing 0.05 or 0.1 mM FA. We selected these low FA concentrations so any changes would be attributed to an effect on fumonisin biosynthesis rather than fungal growth. Indeed, we detected no changes in fungal biomass at these concentrations (Supplementary Table 3). By contrast, 0.1 mM FA caused a significant 50% reduction for FB1 production (*p* < 0.05; Figure 2C).

### Higher Contents of Ferulic Acid in the Pericarp Are Associated With Lower Fumonisin Production

**FIGURE 3:**
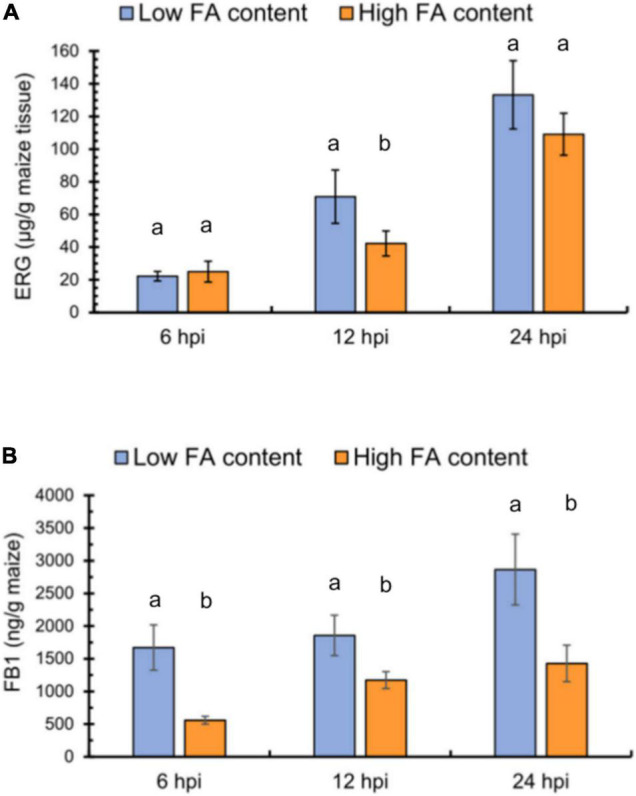
*F. verticillioides* interaction with maize seeds containing low or high ferulic acid levels. **(A)** Fungal colonization, as determined by ergosterol (ERG) levels in maize seeds with low (blue) or high (orange) FA contents at 6, 12, and 24 h post-inoculation (hpi). **(B)** FB1 production in maize seeds with low or high FA contents at 6, 12, and 24 h post-inoculation (hpi). Different lowercase letters indicate significant differences, as analyzed by Student’s *t*-test (*n* = 3; *p* < 0.05).

From the maize genotypes, we separately pooled kernels with low or high FA contents in their pericarp and challenged them by *F. verticillioides* inoculation. We evaluated fungal colonization and FB1 levels at 6, 12, and 24 h post inoculation (hpi) using ergosterol levels to assess the extent of fungal colonization of the seed tissue. The two pools showed no differences in ergosterol contents at 6 hpi, but the LFA pool harbored 30% higher ergosterol levels than the HFA pool at 12 hpi (Figure 3A). At 24 hpi, the two pools no longer exhibited significant differences. However, FB1 levels were consistently lower in the HFA pool, with a three-fold difference observed at 6 hpi and a two-fold difference by the end of the time course (Figure 3B). Because FB1 levels depend on fungal biomass and FA levels, we explored whether an association could be established between these parameters through a bivariate correlation test. Both, fungal colonization, measured as ergosterol levels, and FA content influenced FB1 production (*r*^2^ = 0.79, *N* = 18, *p* ≤ 0.05), being FA levels the variable with major influence (*p* = 0.0001) than ergosterol accumulation (*p* = 0.0004). Moreover, the correlation between FA content and ergosterol levels was not significant (*r^2^* = 0.03, *N* = 18, *p* = 0.443).

### Pericarp Extracts Inhibit *F. verticillioides* Growth

**FIGURE 4:**
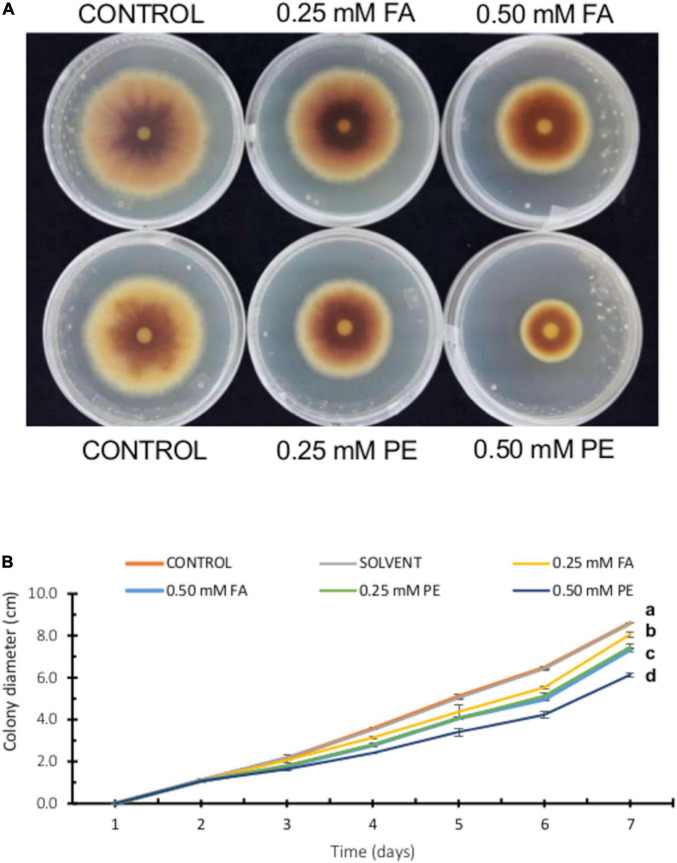
Effect of ferulic acid and pericarp extracts (PE) on *F. verticillioides* radial growth. **(A)** Representative images of *F. verticillioides* growing on PDA plates containing FA (0.25 or 0.50 mM) and pericarp extracts containing the equivalent concentrations of FA. **(B)** Colony diameter of *F. verticillioides* (5 × 10^3^ conidia inoculum) growing on PDA plates containing FA (0.25 or 0.50 mM) and pericarp extracts containing the equivalent concentrations of FA. Values are means ± SD, and different lowercase letters indicate significant differences, as analyzed by ANOVA followed by Tukey’s comparison test (*n* = 3; *p* < 0.05).

To compare our results on FA antifungal activity, we performed a parallel experiment using pure FA and maize pericarp extracts [containing equivalent FA concentrations (0.25 and 0.50 mM)]. As shown in Figures 4A,B, pericarp extracts clearly displayed stronger antifungal activity than pure FA. Although FA is the main phenolic compound in the maize seed pericarp and possesses the highest antifungal activity, other phenolics such as vanillic acid (VA), coumaric acid (CA), and syringic acid (SA) might contribute to the greater antifungal activity observed with pericarp extracts.

### Maize Pericarp Extracts Inhibit Fumonisin B1 Production *in vitro*

**FIGURE 5:**
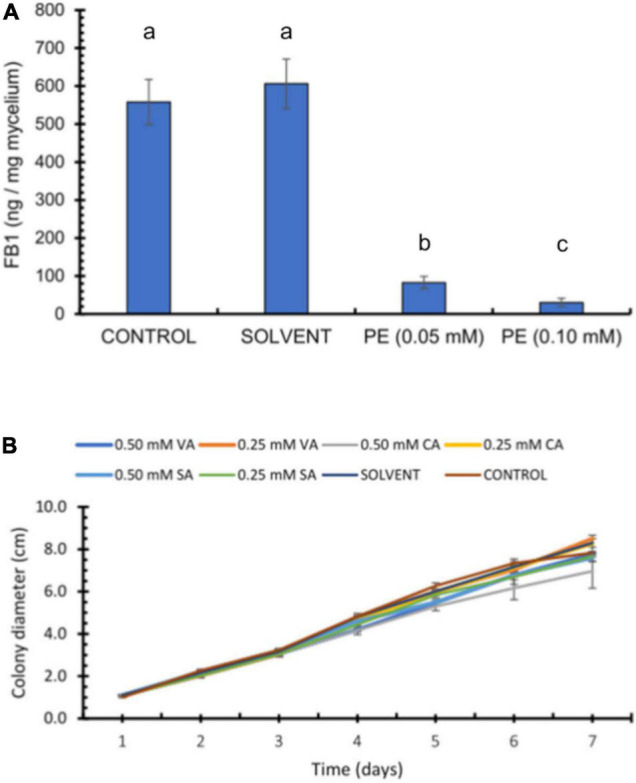
Effect of pericarp extracts (PE) on fumonisin production. **(A)** 1.25 × 10^5^ *F. verticillioides* conidia were inoculated in GYAM medium containing pericarp extracts adjusted to contain 0.05 or 0.1 mM FA. FB1 levels were analyzed by HPLC of the OPA fluorescent derivatives; values are means ± SD, and different lowercase letters indicate significant differences, as analyzed by one-way ANOVA followed by Tukey’s comparison test (*n* = 4; *p* < 0.05). **(B)** Effects of phenolic acids on *F. verticillioides* radial growth. Colony diameter of *F. verticillioides* (5 × 10^3^ conidia inoculum) growing on PDA plates containing vanillic acid (VA), coumaric acid (CA), or syringic acid (SA) (0.25 and 0.50 mM).

To test whether pericarp extracts inhibit FB1 production when added to GYAM medium as previously shown with pure FA, we diluted a methanolic extract from the HFA maize pools to obtain equivalent FA concentrations of 0.05 and 0.1 mM. Indeed, both dilutions of PE disrupted FB1 production by *F. verticillioides* (Figure 5A). The pericarp extract containing 0.05 mM equivalent FA caused an 85% decrease of FB1 levels, while extract with 0.1 mM equivalent FA resulted in a 95% drop in FB1 biosynthesis. As observed with the antifungal assay above, pericarp extract were more potent than pure FA, likely due to the presence of additional phenolic acids, among other compounds. These data were consistent with our finding on the antifungal activity shown by pericarp extracts (Figure 3). We also tested the antifungal activity of several other phenolics and determined that VA, CA, and SA indeed possess antifungal capacity (Figure 5B).

### The Antioxidant Capacity of Pericarp Extracts Is Associated With Ferulic Acid Contents

**FIGURE 6:**
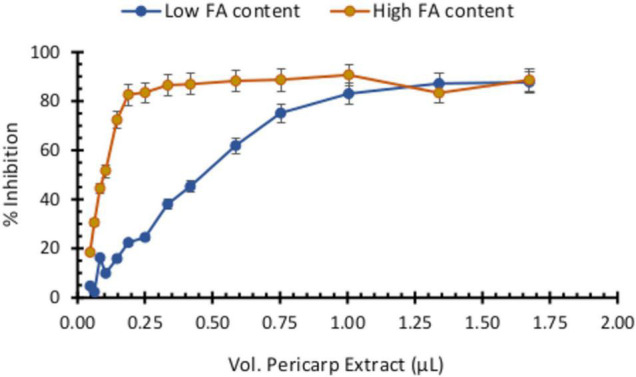
Antioxidant capacity of pericarp extracts (PE) with high or low ferulic acid contents. Antioxidant activity was determined by the ABTS^+^ assay through inhibition of absorbance at 450 nm. Pericarp extracts were prepared to reflect the same amount of biomass (mg pericarp/mL) with increasing volumes of each pericarp extract pipetted into the plate containing the ABTS^+^ reagent.

Because the antitoxigenic activity of several compounds has been associated with their antioxidant capacity, we assessed pericarp extracts for antioxidant potential with HFA and LFA materials using the ABTS^+^ assay. We determined that pericarp extracts from HFA maize varieties possess a greater antioxidant activity than those from LFA genotypes (Figure 6). For this experiment, we prepared extracts so they correspond to the same amount of biomass (mg pericarp/mL extract). Based on a dose–response curve and the pericarp extract volume that resulted in a 90% inhibition of absorbance, we estimated that the titer of this extract is between 0.150 and 0.200 mM FA. Notably, this FA concentration fell within the same range we used for inhibition of FB1 production in our assays (Figure 5B).

## Discussion

We documented a wide variation of esterified FA contents in the seed pericarp among maize genotypes and established a kernel assay to study their interaction with *F. verticillioides*. By comparing the results from this assay with the activity of FA in the inhibition of FB1 production and fungal growth, we established that this phenolic is the main active compound in the maize pericarp. Both pure FA and a pericarp extract containing FA suppressed fungal growth and FB1 accumulation in culture. In kernels inoculated with *F. verticillioides*, both fungal colonization and FA levels influenced FB1 production by comparing maize pools comprising LFA and HFA genotypes, and a significant correlation could be established between FA content and FB1 levels throughout the study period.

We detected a wide range of esterified FA in the pericarp of the 51 maize genotypes studied in this work; levels ranged from 4.5 to 26.3 mg FA/mg dry pericarp tissue. Because we employed an alkaline hydrolysis step during extraction, we reported bound and free FA. We conducted a more detailed analysis on the kernel pools and determined that bound FA represents over 99% of total FA in pericarp tissue. These results agree with those reported by Adom and Liu (2002) who found that in whole maize kernels free FA content represents 0.1% of the total FA. Most of the FA in maize kernels is concentrated in the pericarp tissue; Das and Singh (2015) analyzed the phenolic acid contents in the pericarp from Dent and Flint corn kernels and determined that FA is the most abundant phenolic compound, followed by vanillic acid, syringic acid, protocatechuic acid, coumaric acid, and caffeic acid. FA comprised 40.4 and 48.8% of the total pericarp phenolics in Dent and Flint corn, respectively. Likewise, bound FA in the pericarp represented 44% of total bound FA in kernels and 36.7% of total free FA in kernels. Thus, although other phenolic compounds are present, FA is likely associated with most of properties studied here.

A literature review on the levels of phenolic acids in maize pericarp reveals large differences, with most of these discrepancies possibly due to sample preparation. For example, in the study by Das and Singh (2015), the kernels were milled to separate germ and pericarp from the endosperm using a grain mill, and the maximum reported FA content was 2.85 mg/g. Bily et al. (2003) employed a pearl mill to separate the pericarp-aleurone layer from the endosperm and reported FA levels ranging from 18.53 to 21.9 mg FA/g in seven maize genotypes. Likewise, Sampietro et al. (2013) freeze-dried the maize kernel samples, soaked them for 4 h at 4°C, and milled them. The pericarp tissue was dissected manually using a scalpel, and total FA levels in 11 maize genotypes ranged from 10.7 to 19.9 mg/g, which were similar to the values reported in this work.

Vast differences in the contents of FA and other phenolics can be detected when phenotyping maize varieties and inbred lines. Because the pericarp where phenolic acids are concentrated comprises less than 10% (6.5–9.8%; Zilic et al., 2011) of the total kernel dry weight, these differences may be masked if whole kernels are analyzed. For example, a narrow range of 1.40–1.64 mg total FA/g was reported between 18 maize varieties when the whole kernel was analyzed (López-Martínez et al., 2009).

Several studies have explored the effects of phenolic acids, mainly against *F. graminearum* and *F. verticillioides*. Ponts et al. (2011) tested five phenolic acids in radial growth assays and determined that FA has the highest antifungal activity against *F. graminearum*, followed by coumaric acid, syringic acid, caffeic acid, and *p*-hydroxybenzoic acid. The half maximal inhibitory concentration (IC_50_) values for FA ranged from 0.7 to 2.2 mM, depending on the strain. These results revealed a three-fold difference in IC_50_ values across four strains of the same fungal species. A wider range of concentrations for phenolic compounds has since been tested in assays against *Fusarium* spp., with reported IC_50_ values ranging from 0.7 to 10 mM, depending on the test conditions and fungal strain (Atanasova-Penichon et al., 2016).

Because of these discrepancies and large variations found in the literature, we tested several FA concentrations for growth inhibition of *F. verticillioides* MY3 strain and established that FA concentrations above 2.5 mM completely inhibit fungal colony expansion. We further determined that effective FA concentrations that inhibit *F. verticillioides* development ranged from 0.25 to 1 mM. We optimized the experimental conditions to reduce intra-assay variability by adjusting the titer of the conidial suspension and placing the suspension at the center of a sterile disk on growth medium. These modified conditions produced reproducible results in 7 days (Figure 4) instead of 14 days as in previous assays (Figure 2). These new assay conditions gave us better control to assess the effects of FA concentrations that do not affect fungal growth on FB1 production, using both pure FA and pericarp extracts.

Such technical considerations are relevant, as Ferrochio et al. (2013) evaluated the efficacy of FA (1, 10, 20, and 25 mM) on fungal growth and fumonisin production by *F. verticillioides* and *F. proliferatum* on maize-based medium. They observed that both *Fusarium* species experienced a significant growth reduction at high FA concentrations (10–25 mM), concurrently with decreased fumonisin production. However, lower FA concentrations (1–10 mM) were associated with increased toxin production. At the FA concentrations tested here, we observed no adverse growth effects but did observe a significant decrease in FB1 production in both assays employed. We tested FA concentrations that did not adversely affect fungal growth and observed that 0.1 mM FA inhibits FB1 production by *F. verticillioides* when the fungus was grown on GYAM medium. A methanolic pericarp extract diluted to contain 0.1 mM FA resulted in a more pronounced disruption of FB1 biosynthesis relative to pure FA. As stated above, FA contents are high in the pericarp (40–50% of total phenolics, depending on the genotype), but other compounds likely contribute to this activity as well. Similar results were reported with a wheat (*Triticum aestivum*) bran phenolic extract that contained 0.1 mM FA equivalent and caused a 68% inhibition of trichothecene production by *Fusarium culmorum*, compared to only 21% inhibition from pure FA at the same concentration (Boutigny et al., 2010). Besides phenolic acids found in the pericarp such as vanillic acid and coumaric acid that might contribute to this inhibition, other metabolites might have large effects. For example, low concentrations of α-tocopherol (0.1 mM) inhibited fumonisin production by 30–70% in *F. verticillioides* (Picot et al., 2013). Tocopherol contents are much lower in maize kernels than those of phenolic acids, with levels ranging from 3.9 to 5.4 μg/g (Das and Singh, 2015).

The proposed mode of action of phenolic acids on mycotoxin biosynthesis is thought to require its antioxidant activity. Evidence for this mechanism is strong for trichothecene production by *F. graminearum* and *F. culmorum*. In *F. graminearum*, oxidative stress caused by incubating liquid cultures with 0.5 mM hydrogen peroxide (H_2_O_2_) induced a three-fold increase in the production of two trichothecene mycotoxins, deoxynivalenol and 15-acetyldeoxynivalenol, compared to the non-treated controls. Such accumulation has been shown to be the result of a 20-fold induction in the expression of at least five genes (*Tri4*, *Tri5*, *Tri6*, *Tri10*, and *Tri11*) involved in mycotoxin biosynthesis. Addition of antioxidants such as catalase in the growth medium repressed this transcriptional activation, thus supporting the role of the cell redox status on mycotoxin production (Ponts et al., 2006, 2007). In *F. graminearum*, 0.5 mM FA repressed the expression of *Tri* genes (*Tri4*, *Tri5*, *Tri11*, *Tri10*, and *Tri12*) involved in trichothecene mycotoxin biosynthesis to various levels (50- to 250-fold) (Boutigny et al., 2010). In. *F. culmorum* cultures, 0.5 mM FA inhibited trichothecene production by 78–82% and repressed the expression of seven *Tri* genes. Among the six *Fusarium* strains tested, all were susceptible to inhibition by FA but at different rates, ranging from 17 to 78% inhibition (Boutigny et al., 2009).

Such association between oxidative stress and fumonisin production is not as clear in *F. verticillioides*. Several reports have shown a differential effect depending on the strain tested. When various *F. verticillioides* strains differing in their ability to produce fumonisin were treated with 0.5 or 2 mM H_2_O_2_, fumonisin production was induced by 300% in two strains, but was inhibited by 80% in the three remaining strains. One of the strains responding positively to oxidative stress also showed a significant increase in transcript levels of seven *FUM* biosynthetic genes, while other strains displayed minimal or no changes in *FUM* transcript levels (Ferrigo et al., 2015). This differential response among strains was also observed when challenged with FA; a high-fumonisin producer *F. verticillioides* strain incubated with 1 mM FA induced FB1 production, while the same treatment repressed FB1 production and *FUM* gene expression in a low-fumonisin producer (Ferrigo et al., 2021). In this study, we used a single *F. verticillioides* strain (MY3), classified as a high-fumonisin producer among our strain collection (Sánchez-Rangel et al., 2005; Galeana-Sánchez et al., 2017). Further studies must address the diversity in responses among *F. verticillioides* strains to explain the complex transcriptional response to oxidation stress and regulation of mycotoxin biosynthesis within their role in the interaction with the host plant. Importantly, fungal infection induces an oxidative burst by the plant cells that might stimulate toxin production, as well as antioxidant activities by the fungal pathogen (Montibus et al., 2015).

In a previous study of resistance against fungal colonization and mycotoxin accumulation, the influence of the physical and chemical properties of the kernel was addressed (Santiago et al., 2015). Resistance to Fusarium ear rot was evaluated in field trials with 19 maize inbreds and by measuring kernel FA contents; a significant and negative correlation was detected between the degree of ear rot and FA levels (Assabgui et al., 1993). Other antioxidants, such as anthocyanins, have also been associated with *F. verticillioides* resistance; in a field study of four maize genotypes (three pigmented and one yellow), Bernardi et al. (2018) reported that the variety with the highest antioxidant activity and highest anthocyanin kernel levels showed the lowest FER infection scores. A common constraint in field studies is the ear inoculation technique, as it is usually performed with a pin-bar which bypass husk coverage as a resistance mechanism, but damage some kernels upon penetration. To overcome these technical difficulties, there is a need for other inoculation techniques that would allow the study of pericarp properties in the laboratory. Here, we incubated intact kernels with a *F. verticillioides* spore suspension and evaluated fungal colonization and FB1 production at three time points over a 24-h time course. We established that the HFA maize pool exhibits more limited fungal colonization at early times and lower FB1 production throughout the study period compared to the LFA pool (Figure 3).

Besides phenolic acid levels, other pericarp traits have been associated with FER resistance and fumonisin accumulation. In a 2-year field trial, 12 Dent corn hybrids were evaluated for FER resistance and pericarp thickness; the pericarp of eight hybrids with high or intermediate resistance was thicker than that of the four susceptible genotypes tested (Hoenisch and Davis, 1994). Pericarp lipid composition has also been implicated in resistance against *Fusarium* infection and fumonisin production. In a kernel assay of 14 maize genotypes differing in resistance to fumonisin accumulation, Sampietro et al. (2009) found that dewaxing the pericarp with chloroform results in higher fumonisin accumulation in most genotypes tested. Thus, both physical and chemical properties of the pericarp contribute to FER tolerance/resistance.

Ferulic acid is an abundant compound, both in the maize kernel and in the kernel pericarp. However, it is one of the many metabolites present in this plant tissues. Comprehensive data concerning the metabolome of maize kernels is limited. Rao et al. (2014) detected 210 metabolites in whole mature maize kernels of 14 representative maize genotypes. Also, Xu et al. (2021) identified 208 metabolites using untargeted metabolite profiling technology, and phenolic compounds comprise 8.6% of the identified metabolites. Bernardi et al. (2018) analyzed the phenolic profiles of three maize genotypes and reported 55 phenolic molecules. This type of studies reports the annotated metabolites thus they partially reflect the complexity of the tissue. We are not aware of comparable studies on maize kernel pericarp, but because of its abundance in this tissue and its activity toward *Fusarium* growth and FB1 production, we provide evidence that FA represents a viable marker for phenotypic evaluation of defense mechanisms against FER.

## Data Availability Statement

The raw data supporting the conclusions of this article will be made available by the authors, without undue reservation.

## Author Contributions

JP and MT-H: conceptualization and writing and editing. JP, MT-H, JM-F, and MM-A: methodology and results analysis. JP: funding acquisition. All authors have read and agreed to the published version of the manuscript.

## Conflict of Interest

The authors declare that the research was conducted in the absence of any commercial or financial relationships that could be construed as a potential conflict of interest.

## Publisher’s Note

All claims expressed in this article are solely those of the authors and do not necessarily represent those of their affiliated organizations, or those of the publisher, the editors and the reviewers. Any product that may be evaluated in this article, or claim that may be made by its manufacturer, is not guaranteed or endorsed by the publisher.
