# Supplementary material for: In Search of Resistance Against Fusarium Ear Rot: Ferulic Acid Contents in Maize Pericarp Are Associated With Antifungal Activity and Inhibition of Fumonisin Production
Source: Front Plant Sci. 2022 Apr 8;13:852257. doi: 10.3389/fpls.2022.852257 (PMC9024315; doi:10.3389/fpls.2022.852257)
Supplement: Supplementary file 1 [file Data_Sheet_1.PDF]

Supplemental Material.

Supplemental Table 1. Stock ID and entries for maize genotypes analyzed in this study. The genotypes comprising the low-ferulic acid content (LFA; blue) and high-ferulic acid content (HFA; orange) pools are shown.

Code	Stock ID	Maize Entry	Code	Stock ID	Maize Entry
M32	Jiafa-17	DTMA-180	M13	Jiafa-15	DTMA-176
M17	Jiafa-18	DTMA-196	M38	Jiafa-4	A.F&F.v-50
M18	Jiafa-19	DTMA-197	M22	Jiafa-7	DTMA-55
M16	Jiafa-55	Tropical-23	M11	Jiafa-6	A.f&F.v-57
M10	Jiafa-58	Colombia-3	M8	Jiafa-16	DTMA-178
M31	Jiafa-46	Entomol-25	M5	Jiafa-47	Entomol-38
M9	Jiafa-41	Pathology-44	M25	Jiafa-10	DTMA-107
M7	Jiafa-8	DTMA-69	M26	Jiafa-31	GCP-II-137
M20	Jiafa-12	DTMA-141	M41	Jiafa-68	A.f&F.v-99
M21	Jiafa-39	Pathology-11	M51	Chal	Chalqueño
M42	Jiafa-105	Pathology-25	M27	Jiafa-5	A.f&F.v-56
M43	Jiafa-84	DTMA-282	M12	Jiafa-42	Pathology-69
M45	Jiafa-86	DTMA-285	M14	Jiafa-20	DTMA-205
M33	Jiafa-14	DTMA-158	M23	Jiafa-45	Entomol-24
M3	IYADILPRO	Jamay, Jalisco	M28	Jiafa-38	GCP-III-101
M6	Jiafa-35	GCP-III-70	M50	Jiafa-115	Colombia-35
M34	Jiafa-44	Entomol-17	M19	Jiafa-33	GCP-III-4
M47	Jiafa-87	DTMA-286	M4	Global Seed	Celaya Gto
M46	Jiafa-75	DTMA-27	M37	Jiafa-1	A.f&F.v-5
M30	Jiafa-54	Tropical-16	M49	Jiafa-85	DTMA-284
M35	Jiafa-3	A.F&F.v-49	M40	Jiafa-79	DTMA-140
M29	Jiafa-114	Tropical-9	M36	Jiafa-13	DTMA-148
M48	Jiafa-69	DTMA-1	M39	Jiafa-74	DTMA-25
M1	Zarco	Colima	M2	CP	Colegio Postg
M24	Jiafa-25	GCP-II-2	M15	Jiafa-24	GCP-I-85
M44	Jiafa-64	A.F&F.v-79			

Supplemental Table 2. Pedigree of each maize genotype employed in this study.

Stock ID	Maize Entry	Pedigree
Zarco	Colima	ZR-76
CP	Colegio de Postgr.	HI-2
IYADILPR	Jamay, Jalisco	H318
Glob Seed	Celaya Guanajuato	GSG-103
Jiafa-47	Entomology-38	MIRTC5Am F24-2-1-1-4-2-1-B-B-B-B-B-B-B-B-B
Jiafa-35	GCP-III-70	CML-406-B-B
Jiafa-8	DTMA-69	CLA113-B-B
Jiafa-16	DTMA-178	CLQ-RCWQ106=(CML247 x (CLQ-6203xCL-04321)-B-7-1-2)-B-22-1-1-2-B-B-B-B-B
Jiafa-41	Pathology-44	SM003001RBS-12-1-1-B-B-B
Jiafa-58	Colombia-3	CLA146-B-B
Jiafa-6	A.f & F.v-57	CML-155-B-B
Jiafa-42	Pathology-69	(CL G2309 x ([(P390bcoC3 F191-1-1-1-4-B-B-B-B) x (P73TLC3#-96-3-4-#)]-2-2-3))-1-90-1-B-B-B-B
Jiafa-15	DTMA-176	CLQ-RCWQ103=(CML150xCML254)-B-16-2-2-2-B-B-B-B-B
Jiafa-20	DTMA-205	CL-04934 = (P49C2MH12-5-4xP23C2-11-1)-2-2-2-BBBBBBB-B-B
Jiafa-24	GCP-I-85	P84c3BcxLLTtardAsiaxMIRT F41-2-1-2-2-B-B-B-B
Jiafa-55	Tropical-23	CL-02450Q-B-B
Jiafa-18	DTMA-196	CL-RCY023 = (CL-02439*CML-286)-B-1-2-2-B*8-B-B
Jiafa-19	DTMA-197	CL-RCY007=PIO3011F2-3-5-6-1-B*5-B-B-B
Jiafa-33	GCP-III-4	CML-9-B-B
Jiafa-12	DTMA-141	P502c1#-771-2-2-3-B-1-1xCML-176]F2-1-1-2-3-B-B-B-B-B-B
Jiafa-39	Pathology-11	CML-295-B-B
Jiafa-7	DTMA-55	CLA222-B-B
Jiafa-45	Entomology-24	P390amC3/285x287 F27-2-1-3xMIRTC5Am F24-2-1-1-1-1)-2-1 -B-B-B-B-B-B
Jiafa-25	GCP-II-2	CML-22-B-B
Jiafa-10	DTMA-107	[MBR C6 Bc F395-1-B-#-2-2-B-B-B-B-B/CML312SR]-1-1-B-B
Jiafa-31	GCP-II-137	MIRTC5 Bco F62-2-2-1-1-2-1-B-B-B-B
Jiafa-5	A.f & F.v-56	CML-144 -B-B
Jiafa-38	GCP-III-101	P591c4 1y2 GEN F3-1-1-2-B-B-B-B-B
Jiafa-114	Tropical-9	CLQ-RCWQ26-B-B
Jiafa-54	Tropical-16	CL-RCW95-B-B
Jiafa-46	Entomology-25	P390amC3/285x287 F27-2-1-3xP390amC3/287 F23-1-1-2-B)-1-1 -B-B-B-B-B-B
Jiafa-17	DTMA-180	CML-499=(CL-04345*CL-274)-B-15-1-2-B*6-B-B-B
Jiafa-14	DTMA-158	CML-329/MBR c2 am F14-2-BBBB-B-B
Jiafa-44	Entomology-17	MIRTC4Am F101-B-2-1-B-B -B-B-B-B-B-B
Jiafa-3	A.f & F.v-49	CL-02510-B-B
Jiafa-13	DTMA-148	CML-322-B-B
Jiafa-1	A.f & F.v-5	CML 264 Q-B-B
Jiafa-4	A.f & F.v-50	CL-03618-B-B
Jiafa-74	DTMA-25	[CML444/CML395//DTPWC8F31-1-1-2-2-BB]-4-2-2-2-1-BB-B-B-B
Jiafa-79	DTMA-140	[P44 c8 FS 158-3-2--4-1-B-B X CML-321]F2-38-1-BB-B-B
Jiafa-68	A.f & F.v-99	La Posta Seq C7-F180-3-1-1-1-B-B-B-B-B
Jiafa-105	Pathology-28	CL-RCY003=[CL-00331*v]-3-B-3-2-1-B*5-B-B
Jiafa-84	DTMA-282	DTPYC9-F69-3-5-1-1-B-B-B-B
Jiafa-64	A.f & F.v-79	CML395-B-B
Jiafa-86	DTMA-285	La Posta Seq C7-F153-1-2-1-2-B-B-B-B-B
Jiafa-75	DTMA-27	P502-SRc0-F2-54-2-2-1-B-B-B-B

Jiafa-87	DTMA-286	La Posta Seq C7-F12-2-3-1-1-B-B-B-B-B
Jiafa-69	DTMA-1	[CML444/CML395//DTPWC8F31-4-2-1-6]-2-1-1-1-B*4-B-B
Jiafa-85	DTMA-284	La Posta Seq C7-F153-1-2-1-1-B-B-B-B-B
Jiafa-115	Colombia-35	48169-S3-SEL07A-17A-17B-65/65-B-B

Supplemental Table 3. *F. verticillioides* mycelial biomass in GYAM media supplemented with 0.05 mM and 0.10 mM FA. Data shows mean ± S.D. (n = 4).

**Treatment**	**Fungal biomass (mg)**
Control	52.15 ± 2.34
Solvent	52.35 ± 1.89
0.05 mM FA	52.45 ± 1.98
0.10 mM FA	51.27 ± 1.38
